# Abstracts DGNC

**DOI:** 10.1515/iss-2019-2006

**Published:** 2019-03-20

## DGNC: Neurosurgical pain therapy

### Peripheral Nerve Field Stimulation (PNFS) for Chronic Lumbar Pain and the Predictive Value of Transcutaneous Electrical Nerve Stimulation (TENS) for Patient Selection

(Abstract ID: 37)

F. Schwarm^1^, M. Ott^1^, M. Stein^1^, E. Uhl^1^, M. Kolodziej^1^

^1^*Universitätsklinikum Gießen*

**Background:**

PNFS is an effective alternative treatment for patients with chronic low back pain. TENS is known for easy application and frequently used in multimodal pain therapy concepts. The aim of this prospective study was to examine the predictive value of TENS treatment for later successful PNFS.

**Materials and methods:**

Between 2014 and 2017 a prospective cohort study of 23 patients with chronic lumbar pain was conducted. All patients were treated with multiple pain medications. Pain intensity (NRS), SF12v2 with Physical Component Summary (PCS) and Mental Component Summary (MCS) scores, and Oswestry Disability Index (ODI) were assessed before and after TENS use, pre- and postoperatively as well as 3 and 6 months after PFNS implantation. TENS was applied for 3-5 days. The implantation of a rechargeable PNFS-system with 2 percutaneous leads was performed after 4-7 days of positive testing. Statistical analysis was performed using the Mann-Whitney U and the Wilcoxon rank-sum test.

**Results:**

The cohort consisted of 23 patients (9 females, 14 males) with a median age of 60.9 years (IQR25-75 52.6-65.7). After positive PNFS testing a neurostimulator was implanted in 8 patients with positive TENS effect and 10 patients with no TENS effect. 5 patients had no PNFS effect and the test electrode was explanted. After 3 and 6 months median NRS was reduced from 8.5 (IQR25-75 7-9) to 2.5 (IQR25-75 0-3.8; p=0.001) and 3.0 (IQR25-75 2.3-3.8; p=0.001), ODI from 57.0 (IQR25-75 46.5-66) to 38.0 (IQ IQR25-75 19-51; p=0.01) and 34.0 (IQR25-75 15-49; p=0.01) in the TENS positive cohort, respectively. The TENS negative cohort showed a median NRS reduction from 7.0 (IQR25-75 6-8) to 5.0 (IQR25-75 3-6.5; p=0.010) and 4.0 (IQR25-75 3.0-7.3; p=0.018), after 3 and 6 months, respectively. There was no improvement from initial ODI 45.0 (IQR25-75 44.0-62.5) neither after 3 nor after 6 months. Generic health survey scores (PCS and MCS domains of the SF-12v2) showed slightly better improvement in the TENS positive cohort. All patients with negative PNFS testing belonged to the TENS negative cohort.

**Conclusion:**

TENS positive patients showed a significantly better treatment effect after PNFS implantation, thus TENS can be a predictive tool for patient selection in PNFS. PNFS itself is an effective and safe treatment for chronic low back.

### Adequate and individual pain regimes are important especially after long spine surgeries

(Abstract ID: 157)

T. Fortmann^1^, W. Stummer^2^

^1^*Hamm*

^2^*Uniklinik Münster*

**Background:**

Patients suffer from pain after surgery due to transsection and suturing of skin and other tissues. The aim of this study was to introduce a suitable pain medication management by improving nurses’ autonomy and thereby reducing patients’ pain.

**Materials and methods:**

During three time periods from December 2011 until May 2014 a total of 275 patients (112, 83, 80) were interviewed using the QUIPS-questionnaire to register pain in different situations during the first 24h after surgery, the dose of pain medication, side effects and patients’ satisfaction. Between the first and the second period all doctors and nurse were introduced to the Targin and Palladon-schemes. We also differentiated between head and spine surgery and analysed the key parameters of the performed surgeries.

**Results:**

Although there was no significant difference between the three groups concerning different kinds of pain, patients’ satisfaction after head surgery rose significantly from 11,85 to 12,83 (p=0,004). Unfortunately these patients suffered more from nausea and vomiting (18 vs 5%, p=0,003).

There was a significant difference (< 0,1%) between head and spine surgery in minimal pain (1,58 vs 2,43), pain during mobilization (3,69 vs 4,92) and the reduction of mobility (40 vs 71%).

Especially patients with spine surgery lasting more than 2 hours had more pain during mobilization (5,39 vs 4,21, p=0,032), more requests for pain medication (23 vs 2%, p=0,002) and woke up more frequently due to pain (60 vs 34%, p=0,011).

Interestingly those patients, who received opioids in the anesthetic recovery room had far more pain problems (reduced mood 44 vs 23%, maximum pain 6,18 vs 4,52 (p<0,1%), minimum pain 2,36 vs 1,67, pain during mobilization, 4,73 vs 3,87, more request for pain medication 25 vs 10%, waking up due to pain 57 vs 37%, reduced mobility 64 vs 48% and less satisfaction 11,28 vs 12,03 (p<1%)).

**Conclusion:**

Using pain medication schemes helps to improve patients’ satisfaction since nurses are able to administer more pain medication in pre-determined ranges. For patients after head surgery it might be necessary to reduce the pain medication in order to reduce side effects such as nausea and vomiting. Patients after spinal procedures of longer than two hours should receive enough pain medication right away to improve their mobility and satisfaction.

### Selective L4 dorsal root ganglion stimulation evokes pain relief and changes of inflammatory markers: Part I profiling of saliva and serum molecular patterns

(Abstract ID: 193)

T. Kinfe^1^

^1^*Universitätsklinikum Bonn*

**Background:**

Total knee arthroplasty (TKA) due to osteoarthritis and especially after revisions of previous TKA have been reported to lead to a refractory chronic post-surgical pain (CPSP). The incidence of CPSP ranges between 10-34% and additionally impact quality of life, mood, sleep, cognition and metabolic state of the affected subjects. Complex regional pain syndrome I - II (CRPS) represent clinical phenotypes of CPSP of the knee region. Complex regional pain syndrome (CRPS) and associated co-morbidities have been linked to a pro-inflammatory state driven by different mediators. Most recently, an approach that appears to have a considerable promise for treating focal neuropathic pain has become available (dorsal root ganglion stimulation; DRGSTIM). Anatomically targeted DRGSTIM was found to be superior to conventional SCS in a Class I study as well as several controlled and uncontrolled observational clinical trials for a variety of pain conditions. Briefly, DRGSTIM may have the capability to restore the distorted filter function of the DRG, thus inhibiting hyperexcitability of DRG neurons and deeper layer compartments (laminae II/III) of the spinal cord. The precise mechanism of DRG-evoked effects on spino-nociceptive neural transmission as yet is not fully established. However, clinical trials assessing the impact of DRG stimulation on the neuro-immune communication are lacking.

**Materials and methods:**

The goal of this study was to assess concentration changes of neuroinflammatory mediators in serum [interleukins (IL-1β, IL-6, IL-10), tumor-necrosis factor (TNF-a), high-mobility group box 1 (HMGB-1), brain derived neurotrophic factor (BDNF), leptin, adiponectin, ghrelin] and in saliva [oxytocin; OXY] relative to selective L4-DRG therapy.This study enrolled 24 subjects (12 refractory CRPS patients plus suitably matched healthy controls) and performed immunoassays of inflammatory mediators in saliva and serum along with score-based assessments of pain, mood and sleep quality at baseline and after 3 months of selective L4-DRGSTIM.

**Results:**

After 3 months L4-DRGSTIM CRPS associated pain significantly decreased. In addition, disturbed sleep and mood improved post-DRGSTIM, although statistically not significant. Significantly increased serum values of pro-inflammatory markers were detected pre- and post L4-DRGSTIM for high-mobility group box 1, tumor-necrosis factor α, interleukin (IL) 6 and leptin. IL-1β was significantly elevated pre-L4 DRGSTIM, but not post-treatment. Elevated anti-inflammatory IL-10 significantly decreased after 3 months in serum, while saliva oxytocin concentrations increased in CRPS subjects after L4-DRGSTIM (p=0.65). No severe implantation and stimulation associated adverse events were recorded.

**Conclusion:**

Selective L4-DRGSTIM improved neuropathic pain and functional impairment in CRPS as previously reported. CRPS patients displayed a pro-inflammatory molecular pattern in serum. Serum anti-inflammatory IL-10 significantly declined, while saliva oxytocin non-significantly increased after L4-DRGSTIM. An evidence-based relational interpretation of our study is limited due to the uncontrolled study design. However, molecular profiling of biofluids (saliva, serum) represents a novel and experimental field in applied neuromodulation, which warrant further investigations to unveil mechanisms of neuro-immune modulation.

**Picture:**
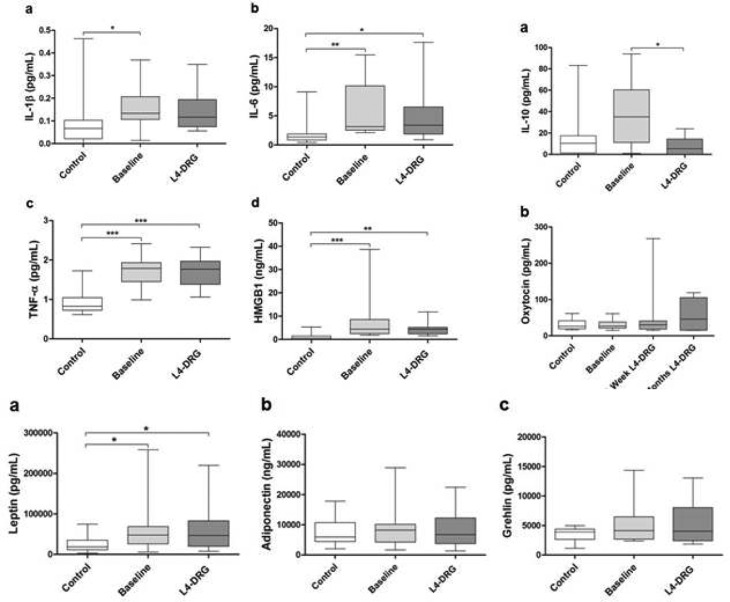
Interleukin-1ß, interleukin 6, TNF, high-mobility group box 1 protein (HMGB1), Adipokine peptides, IL-10 and oxytocin serum analysis. A comparison of baseline assessment and after 3 months selective L4-DRGSTIM (two right columns) compared to those of healthy controls (HC).

### Retroperitoneal Sacral Nerve Stimulation for the Management of Chronic Pelvic Pain After Endometriosis Treatment

(Abstract ID: 335)

F. Schwarm^1^, H. R. Tinneberg^1^, E. Uhl^1^, M. Kolodziej^1^

^1^*Universitätsklinikum Gießen*

**Background:**

Endometriosis or surgery for it can result in secondary nerve damage and chronic pelvic pain. The results of conservative, surgical treatment, and spinal cord stimulation show only a poor outcome. We present a new surgical method of retroperitoneal sacral nerve stimulation for the treatment of neuropathic pelvic pain caused by endometriosis.

**Materials and methods:**

Between 2012 and 2017 five female patients were retrospectively analyzed. All were complaining of chronic pelvic pain, and bladder and bowel dysfunction following several surgeries for endometriosis. These patients underwent laparoscopic re-exploration or re-laparotomy with retroperitoneal implantation of electrodes directly onto the sacral nerve. The intervention was followed by a test period (3-15 days) before the decision for a permanent implantation of a neurostimulator was made. Pain intensity (NRS), generic health status (EQ-5D-5L), Becks Depressions Inventary (BDI-V) and Pain Catastrophizing Scale (PCS) were assessed for the immediate pre- and postoperative status as well as after 3 and 6 months postoperatively. Statistical analysis was performed using Mann-Whitney U and Wilcoxon rank-sum test.

**Results:**

Median age was 40.1 years (IQR25-75 29.4-47.0). Significant improvement of NRS from median 9.0 (IQR25-75 8.5-10.0) preoperatively to 5.0 (IQR25-75 1.75-6.25; p=0.01) at 3- and 2.5 (IQR25-75 0.25-4.5; p=0.01) at 6-month follow-up was achieved. Median EQ-5D-3L index value before treatment was 0.19 (IQR25-75 0.13-0.31) indicating a low quality of life. After 3- and 6-months a significant improvement to 0.73 (IQR25-75 0.71-0.90; p=0.04) and 0.76 (IQR25-75 0.74-0.95; p=0.03) was seen. The preoperative median BDI-V score of 46.0 (IQR25-75 40.5-58.0) indicating a major depressive mood increased significantly improved to 26.0 (IQR25-75 19.0-39.0; p=0.0264) and 12.0 (IQR25-75 4.5-34.0; 0.0271) after 3 and 6 months respectively. Preoperative PCS was highly elevated with a median score of 42.0 (IQR25-75 37.5-50.5). After 3 and 6 months a significant reduction to 18.0 (IQR25-75 8.0-22.5; p=0.0091) and 3.0 (IQR25-75 1.0-10.5; p=0.0088) was seen, respectively. Furthermore, 4 patients had a complete reduction in demand for analgesics.

**Conclusion:**

This unique method is an effective treatment option for chronic, neuropathic pelvic pain after endometriosis treatment. However, this method requires an interdisciplinary surgical team approach.

## DGNC: degenerative cervical spine diseases

### Diffusion tensor imaging is an alternative to CT-myelography for surgical planning in patients with multilevel cervical spondylotic myelopathy

(Abstract ID: 26)

K. Schöller^1^, S. Siller^2^, C. Brem^2^, J. Lutz^2^, S. Zausinger^2^

^1^*Universitätsklinikum Gießen*

^2^*Uniklinik München*

**Background:**

Despite its invasiveness, computed-tomography myelography (CT-M) is still considered to be superior to conventional magnetic-resonance imaging (MRI) for preoperative evaluation of multilevel cervical spondylotic myelopathy (MCSM). We analyzed if diffusion-tensor imaging (DTI) could be a less-invasive alternative for this purpose

**Materials and methods:**

In 20 patients with MCSM and indication for decompression of at least one cervical level, CT-M was carried out preoperatively to determine the extent of spinal canal / CSF space and cord compression (Naganawa score) for decision on the number of levels to be decompressed. Fractional anisotropy (FA) and apparent diffusion coefficient (ADC) as the main DTI indices were correlated with these parameters and with MRI-based increased signal-intensity (ISI); ROC-analysis was performed to determine the sensitivity to discriminate levels requiring decompression surgery. European-Myelopathy-Score (EMS) and neck/radicular Visual-Analogue-Scale (VAS-N/-R) were utilized for clinical evaluation.

**Results:**

According to preoperative CT-M, 20 levels of maximum and 16 levels of relevant additional stenosis were defined and decompressed. Preoperative FA and particularly ADC showed a significant correlation with the CT-M Nagawana score but also with the ISI grade. Furthermore, both FA and ADC facilitated a good discrimination between stenotic and non-stenotic levels with cut-off-values being < 0.49 for FA and > 1.15x10-9 m2/s for ADC. FA and especially ADC revealed a considerably higher sensitivity (79% / 82%) in discriminating levels requiring decompression surgery compared to ISI (55%). EMS and VAS-N/-R were significantly improved at 14 months compared to preoperative values.

**Conclusion:**

In MCSM patients, DTI parameters are highly sensitive to identify levels requiring surgical decompression and might therefore represent a less-invasive alternative to CT-M for surgical planning.

### Successful conservative treatment of a bilateral C5 palsy following posterior cervical decompression and fusion surgery for ossification of posterior longitudinal ligament

(Abstract ID: 66)

S. W. Kim^1^, M. Bender^1^, F. Gronen^2^, E. Uhl^1^, K. Schoeller^1^

^1^*Universitätsklinikum Gießen*

^2^*Diagnostikzentrum Radiologie+Neurologie, Gießen*

**Background:**

Ossification of the posterior longitudinal ligament (OPLL) is a relatively rare cause of cervical spinal canal stenosis and myelopathy in Central Europe. In cases of severe or progressive myelopathy symptoms, surgical decompression of the spinal cord might be indicated, usually through a posterior approach. C5 palsy (C5P) is a well-known complication of anterior and posterior cervical decompression, particularly in multilevel surgeries that involve the level C4/5. Most of the palsies occur unilaterally. Bilateral cases, however, are very rare, and only few reports have previously described their outcome. We report about a patient with a severe progressive bilateral C5P after posterior decompression and fusion surgery for OPLL who was closely followed by 3 monthly clinical and neurophysiological examinations. The patient has almost recovered complete strength at 12 months under conservative therapy in adherence to a rigorous physical therapy protocol.

**Results:**

Casereport

A 58 year old female patient with ossification of the posterior longitudinal ligament and resulting spinal canal stenosis underwent surgery in August 2015. Weakness in her both deltoid and biceps muscles was immediately detected after surgery and gradually deteriorated to severe paresis in the following 2 days. Postoperative magnetic resonance imaging showed sufficient decompression of the spinal cord with posterior shifting. However, residual neuroforaminal bilateral stenosis at levels C4/5 and C5/6 was found on postoperative computed tomography. After discussion of the therapeutic options, we decided to start conservative management including physical therapy and to follow the patient closely including clinical and neurophysiological examinations for the next 12 months. After 3 months, improvement of bilateral paresis was already evident. Muscle strength in the right biceps and the right deltoid muscles recovered completely within a year, but Grade 4 muscle weakness remained in the left deltoid and biceps muscles. Repetitive electromyography also showed continuous improvement of the interference patterns of the deltoid and biceps muscles.

**Conclusion:**

Several factors potentially influence the development of a C5P after cervical decompression but, up to know, no surgical strategy offers clear advantages over the other. Posterior shifting of the spinal cord should be avoided, but this is not always possible. Nevertheless, the prognosis of a C5P is good, and in our opinion, physical therapy using a rigorous treatment protocol is also adequate in patients with severe and bilateral palsies.

### Double level Hybrid surgery of the cervical spine – combination of ventral fusion and artificial disc implantation on top vs double level fusion by PEEK Cages stand alone

(Abstract ID: 364)

S. Gitter^1^

^1^*Nova-Clinic, Biberach*

**Background:**

The author conducted a retrospective study to compare the implantation of polyetheretherketone (PEEK) cages stand alone after anterior cervical discectomy for double level cervical degenerative disc disease to the hybrid surgical procedures by implantation of one level peek cage in combination with artificial disc implantation on top.The significant importance of pseudarthrosis and impact pathology in stand alone PEEK cage implantation procedures is well discribed in the literature.

**Materials and methods:**

Comparison oft wo gropus: 80 patients double level classical stand aone cage vs 80 patients lower level fusion and artificial disc on to level.Preoperative and postoperative radiological and clinical assessments were typically performed.

**Results:**

During the mean follow-up period of 36 months, fusion occurred in 73% of patients in the stand alone group. Subsidence developed in 37% oft he patients in the double level stand alone PEEK cage implantation cohorte, clinical relevant in lower than 8%. Subsidence developed in 25% oft he patients in the cage plus artificial disc group, clinicl relevant lower than 5%.Significant pain relief in the first 24 month, related tot he myelon- and root decompression.In the further follow up we observed significant axial , facet related, pain in the double level fusion group in 45% vs 20% facet related pain in the cage plus artificial disc group.In the follow up period we observed relevant , intermittend adjacent level disc desease in 9% oft he PEEK cage stand alone group and in 2.5% oft he cage plus artificial disc group.The subsidence rate is associated further with cage hight (>6mm).Rate of pseudarthroses: in the cage stand alone group: 15% , in the cage plus artificial disc group: 2%We did not observed any insufficiency or dislocation oft he artificial disc during follow up, no heterotopic ossification in the artificial disc level. The rate of adjacent level pathology is significantly lower in the group of fusion with PEEK cage in combination with artificial disc implantation on top.

**Conclusion:**

There is in all parameters a significant advantage of PEEK cage implantation in combination with artificial disc implantation on top vs the group of double level fusion by Peek cages in stand alone technique.Fusion and / or subsidence affect the clinical outcomes. Using of artificial disc on top in combination with a lower level PEEK cage implantation is an effective option (recommendet) in cases of combined doubel level disc degeneration.

### Stabilzation of the cervical spine using a dynamic plate (spanning over 2 levels) vs two level cage Implantation

(Abstract ID: 365)

S. Gitter^1^

^1^*Nova-Clinic, Biberach*

**Background:**

The author conducted a retrospective study to compare the implantation of polyetheretherketone (PEEK) cages after anterior cervical discectomy for double level cervical degenerative disc disease to the additional dynamic ventral screw and plate fixation in 2 different systems (2 different polyaxial screw systems). The significant importance of pseudarthrosis and impact in stand alone PEEK cage is well discribed.

**Materials and methods:**

A group including a total of 80 patients with double-level degenerative disc disease was treated with anterior cervical discectomy and implantation of PEEK stand-alone cages. We compare this group to another group of 80 patients: the procedure of peek cage implantation was added by ventral dynamic plate and polyaxial screw fixation. Preoperative and postoperative radiological and clinical assessments were performed.

**Results:**

Follow-up period of 36 months: fusion occurred in 73% of patients in the stand alone group and in 96 % of all patients in the "plate added group". Subsidence : 37% of the patients stand alone PEEK cage implantation cohorte. No subsidence in the group of additional platement observed. In the early follow up (up to 24 month) pain decreased in all patients, and the patients' satisfaction rate:79% in the stand alone group vs 92 % in the platement group. Neither fusion or subsidence was related to the clinical outcome in the early time of 12 month. In the further follow up we observed significant axial pain in the subsidence group (56%) vs a facet releated pain (intermittend treatment) in the additional platement group (32%). We observe relevant , intermittend adjacent level disc desease in 9% oft he PEEK cage stand alone group and in 10.5% oft he addional platement cohorte.The history of development of NDI is related tot he rate of axial and facet pain syndromes.The subsidence rate is associated further with cage hight (>6mm).Rate of pseudarthroses in the cage stand alone group: 15%, no pseudarthrosis in the platement group.

**Conclusion:**

The clinical and radiological demonstrate significant difference in outcome between the cage stand alone vs the additional platement- groups. There ist in all parameters a significant advantage in PEEK cage implantation in duoble level procedures in combination with addional plate and screw fixation. Fusion and / or subsidence affect the clinical outcomes. Using of additional platement is an effective option (recommendet) especially in cases of delordotic and kyphotic cases.

### Safe surgical adjunct- and salvage-techniques for posterior atlanto-axial fixation – A retrospective mini case series

(Abstract ID: 580)

M. Schomacher^1^, P. Reid^2^, J. Osorio^2^, A. Jödicke^1^, C. Ames^2^

^1^*Vivantes Klinikum Neukölln, Berlin*

^2^*University of California San Francisco - UCSF*

**Background:**

The atlanto-axial region is a complex anatomical area. Standard posterior fixation techniques (e.g. Goel-Harms-Melcher; Magerl) are used widely. Rare instances (spinal dysplasia, anterior non-fusion, degenerative deformity with anatomical deformation of landmarks) may present challenges in screw placement, achievement of bony fusion and avoidance of vascular injury. Reliable salvage techniques and modifications are helpful if well-established C1-C2 fusion techniques cannot consistently performed.

**Materials and methods:**

Three cases of C1-C2 posterior fusion with necessity for technical modifications of stabilization were reevaluated:

Case 1:

A 76 year-old woman with history of prior C4-C7 ACDF and posterior spinal fusion for cervical myelopathy revealed in MRI a pseudotumor and stenosis of the C1-C2 complex as a result of microinstability. Surgery of the patient was performed over a dorsal cervical midline incision. The prior instrumentation was removed at the upper level at C4. The C3-lamina and C2-pedicles were exposed. On right side a C1-lateral mass screw and C2-pedicle screw were placed. Because of degenerative changes at left side with complexity of C1/C2-articular surface a transarticular C1-C2 screw was placed under navigational assistance. The rods were reattached and decompression of the C1-C2 laminar bone was performed. After decortication from C1 to C5 allograft bone mix with BMP was placed.

Case 2:

A 19 year-old female patient with recurrent symptoms of cerebellar dysfunction demonstrated signs of cerebellar insults in imaging diagnostics (CT, CT-A, MRI), a C1-assimilation anomaly with instability of the C1/C2-segment and a Klippel-Feil-Syndrome at level C4/5. Angiography demonstrated right sided blood-flow reduction at the V3/4-segment during head rotation. Since simple decompression of the vertebral artery was not feasible, occipital-cervical-fusion was indicated. Because of C1-assimilation and dysplastic changes a right C2 lateral mass screw and C2-lamina screw were inserted under navigational assistance. Lateral mass screws were placed at left C3- and C4-level and right C4- and C5-level. Screws were connected with bended rods and fixed to an occipital plate. For supportive boney fusion, patient’s iliac crest material and ceramic bone graft was additionally placed at C1-C2 region.

Case 3:

An 84 year-old woman showed signs of C2 non-union (CT scans) and symptomatic instability (neck pain) after 8 weeks conservative treatment of a C2 fracture (Anderson-D'Alonzo type II). Posterior C1-C2 fixation was performed over dorsal midline incision and exposure of the C1 lateral mass and the C2 pars- and pedicle region. Two C1 lateral mass screws were inserted under navigational guidance. At C2-level pars screws were inserted and fixed to C1-screws with rods. For additional fusion and stabilization a single midline iliac autograft was placed after decortication of the C1 and C2 arch between the C1 lamina and the spinous process of C2 and fixed with a single sublaminar cable.

**Results:**

After surgery all patients showed no new neurological findings and resolved preoperative complaints. Postoperative images (X-ray/CT-Scans) showed a good cervical alignment, regular placed implants, and bony fusion in all cases.

**Conclusion:**

The indicated technical modifications of different C1-C2 posterior stabilization procedures caused by complex degenerative and anatomical reasons appear to be safe and feasible options. Such type of modified procedures should be considered in each spine surgeon’s surgical preparations. However to proof reliability of such alternative techniques further studies with large numbers of patients should be conducted.

## DGNC: Petrous bone tumors

### Near-field electrodes are the new gold standard for intra-operative evoked potential monitoring of hearing in surgery around the petrous bone

(Abstract ID: 40)

S. K. Rosahl^1^, J. Lehmberg^2^

^1^*Helios-Klinik Erfurt*

^2^*Städtisches Klinikum München GmbH*

**Background:**

Compared to far-field registration, near-field recording of cochlear and brainstem auditory potentials significantly reduces acquisition time during surgery around the cochlear nerve.

We have designed and tested new tympanic and brainstem electrodes to monitor the functional integrity of the lower auditory pathway.

**Materials and methods:**

Stainless-steel, ball-shaped electrodes were developed in co-operation with inomed Medizintechnik GmbH (Emmendingen, Germany). The first of these electrodes was connected to a stainless-steel spring running through an insert earphone. It attaches to the tympanic membrane to record a peripheral auditory evoked potential (ECoG). A second mini ball electrode with a diameter of 1.6 mm was placed either on the cochlear nerve or at the dorsal cochlear nucleus of the brainstem to record a central auditory brainstem response (CABR).

Dual near-field recording (DNF) with both electrodes in place was applied in a series of 589 patients with lesions in the CPA and internal auditory canal during microsurgery. Near-field potentials have been compared to the far-field Auditory Brainstem Response (ABR, Cz-A1/A2, band-pass 100 - 3000 Hz). Click stimuli were applied at 13.2 Hz. Data processing was carried out with a commercially available electrophysiological monitoring system (ISIS®, inomed). Waveforms were injected into the visual field of the operating microscope.

**Results:**

Waves I and II of the BAEP were best identified in recordings at the tympanic membrane, wave III to V were largest in recordings at the brainstem or with electrodes places on the cochlear nerve. Compared to far-field recordings, amplitudes were 3 - 4 times larger for ECoG, and 2 - 20 times larger for CABR in patients with functional hearing. CABR was more variable than the ECoG, depending mostly on electrode location relative to the brainstem generators. In patients with good pre-operative hearing acquisition time for reliable averages were 40 seconds in far-field, and as low as two seconds in near-field recordings.

**Conclusion:**

Non-invasive electrodes have rendered near-field recording of electrophysiological responses to auditory stimuli the gold standard in hearing-preserving surgery in lesions around the cochlear nerve.

In lesions located intradurally around the petrous bone, dual near-field recording adds safety and significantly shortens feedback time to the surgeon, especially with video microscope injection of waveforms.

**Picture:**
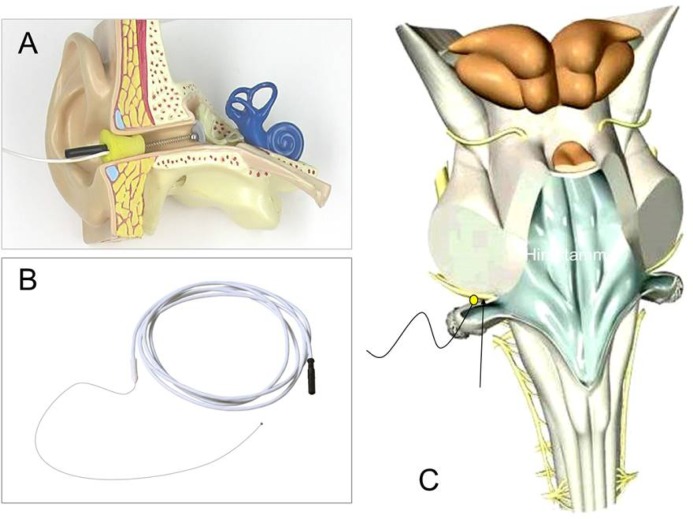
Fig.1 ECoG electrode in the outer ear canal (A), CABR mini-ball electrode (B), and recording site near the brainstem entry zone of the cochlear nerve

### Treatment of Vestibular Schwannoma – the interdisciplinary way

(Abstract ID: 737)

R. Behr^1^, K. Schwager^1^, E. Hofmann^1^

^1^*Klinikum Fulda*

**Background:**

The treatment of vestibular Schwannomas (VS) changed over the last decades. Initially patients were diagnosed mostly with large tumors. The operation was the only treatment modality. With availability of CT and MRI, diagnosis was made earlier and treatment strategies changed. Nowadays 3 modalities are accepted: wait and scan, microsurgical operation and radiotherapy. We add a forth, the interdisciplinary counseling in a skull base center and operation by an oto-and neurosurgical team. This "forth way" provides an optimum in pretherapeutic management and counseling by a peer group of neuro- and otosurgeons, neuroradiologists, neurologists, oncologistc, radiotherapists and an experienced team of neuro- and oto-surgeons, performing the operation together. This concept was started in 1992 at Würzburg University "Kopfklinik" and continued by the authors at different institutions since now.

**Materials and methods:**

125 patients, including 7 recurrencies (5.6%), were retrospectively analyzed. The operative concept is a retrosigmoid approach in semi sitting position. IOM was intensively used by SSEP, MEP, direct facial monitoring, facial MEP and BERA. The extrameatal tumor was removed by the neurosurgeon and the intrameatal by the otosurgeon. All tumor sizes and recurrencies were evaluated, only intrameatal location was excluded. The House-Brackmann (HB) scale was slightly modified as to grade 1 comprises HB 1 and 2, grade 2 HB 3 and grade 3 HB 4-6. For hearing evaluation the Gardner-Robinson (GR) scale was used.

**Results:**

Of all tumor sizes, 7 patients (5.6%) had a grade 3 facial paresis, most of them with large tumors and recurrency. 116 patients (92.8%) had grade 1, excellent to good results, HB 1-2, one year after the operation. Only a few patients were in grade 2, equivalent HB 3. In all but 2 recurrent tumors the HB grade remained the same as preoperatively. In 3 grade 1, in 2 grade 2 and in 1 grade 3. Facial outcome was related to extrameatal tumor size. In tumors larger than 3.1 cm diameter facial function was in 23% grade 3. Also the rate of hearing preservation was clearly related to tumor size. In 88 patients, 70.4%, there was a preoperative hearing according to GR of 1-3. Which means not in every case a servicable hearing but an augmentable hearing with appropriate hearing aids. Over all tumor sizes the postoperative hearing was in 42 patients, 47.8%, GR 1-3. An interesting parameter was the feasibility of reproducible BERA recordings. In patients with extrameatal tumors of up to 20mm and a preoperative hearing of GR 1-2 and reliable recordings of BERA, the hearing outcome in the same GR scale of 1-2 was 68% (15/22). Including patients with the same Tumor size and preop hearing but worse BERAs, the result was 53.84% (21/39). There were 5 subctaneous CSF pouches and 2 liquorrhea, 1 superficial wound infection and one transient hemiparesis due to pneumatocephalus.

**Conclusion:**

The interdisciplinary treatment of VS provides a safe and effective treatment of this disease. The rates of facial nerve and hearing preservation are in the same range as in large other contemporary studies. If the BERA recordings are technically stable and free from artefact, excellent rates of hearing preservation were obtained. A highly sophisticated intraoperative monitoring is essential for good results. With our setup, the learning effect for both surgeons is maximized and the intraoperative discussion is fruitful for the operative team, the students, nurses as well as for the patients outcome. In addition, it bears an excellent possibility to simultaneously train younger skull base specialists from both fields.

### Prosthetic hearing with ABI in NF-2: Aspects for improved outcome

(Abstract ID: 758)

R. Behr^1^, K. Schwager^1^, E. Hofmann^1^

^1^*Klinikum Fulda*

**Background:**

ABI is a generally accepted method to restore hearing after destruction of the cochlear nerve mostly in neurofibromatosis type 2 (NF-2) patients. In the early years however speech understanding was not possible especially not without visual cues as lipreading. Newer data in NF-2 showed that despite of large tumors a considerable percentage of patients achieved open set speech recognition and even telefone use. In this retrospective study we tried to find factors associated with higher levels, better than 30% open set sentence recognition, of prosthetic hearing.

**Materials and methods:**

All NF-2 patients from 6 ABI centers implanted from 1997-2011 with a Med-El ABI device and with at least 3 months of implant experience were evaluated. Only patients out of this group who scored better than 30% open set sentence recognition were investigated more closely. Surgical factors (the author is the main surgeon), patient and tumor characteristics, intraoperative electrophysiology, electrode placement, number of auditory electrodes and distinct pitches, device factors, stimulation strategy and fitting factors were studied.

**Results:**

Of 84 patients 26 scored better than 30% open set sentence recognition (31%). Neither patient age, tumor size or -stage influenced hearing outcome significantly. If deafness before implantation was less 1 year scores were over 65% sentence recognition compared to 45% (p=0.03). Patients operated in semi-sitting position scored significantly better than in lying position (p=0.04). 35.7% of semi-sitting vs 16.7% of lying operated patients scored better than 30%. MCL levels of less than 28nC correlated sign. better , 67.1% correct sentence recognition vs 47.2, p=0.04. Number of audible electrodes had no significant effect on outcome. However patients with average number of distinct pitches of 9.1 had scores over 60% and those with 7.4 had between 30-60% (p=0.01). In patients with high stimulation rates, 1200ppse, scores were 67.1% vs 45.7% in those below 1200ppse (p=0.03).

**Conclusion:**

In this selected group of excellent and top performing ABI patients it was possible to distinguish some parameters which are probably responsible for improved outcomes. The message is, that exact probe positioning is mandatory. Intraoperative mapping of the cochlear nucleus area by test electrodes and painstaking electrophysiological measurements will support optimal placement. This will cause low thresholds and MCL levels, high stimulation rates and many distinct pitches. Surgery must be as meticulous and atraumatic to the nervous tissue as possible, which is facilitated by semi-sitting position. If any possible, timing of implantation should not be postponed longer than one year after deafness.

### Exclusively endoscopic resection of tumors of the cerebello pontine angle

(Abstract ID: 761)

R. Behr^1^, K. Schwager^1^, E. Hofmann^1^

^1^*Klinikum Fulda*

**Background:**

Endoscopic techniques took over an important place in surgery of skull base lesions. In the last 10 to 20 years significant steps forward could be achieved in surgical treatment of anterior skull base lesions. Especially pituitary adenomas and, with the extended approaches, meningeomas and craniopharyngeomas are now successfully treated with exclusively endoscopic approaches. In the posterior fossa the use of the endoscope is still limited. It is mostly used for exploration of hidden places, which are difficult to be disclosed by the microscope. The question therefore arises if the endoscope can be used more intensively also in lesions of the posterior fossa.

**Materials and methods:**

In a prospective analysis 8 female and 2 male patients (pat), with 2 meningeomas (M) and 8 vestibular schwannomas (VS) were operated only by using the endoscope as visualisation tool. A two dimensional Storz endoscope with 0 and 30 degree optics was used. The tumors should not exceed 20mm in extrameatal diameter. VS grew intra- and extrameatal, the M were located laterally to the IAC. Intensive intraoperative monitoring was used. The pat were positioned laterally with slightly elevated head. Follow up period is meanwhile between 42 and 100 months. All pat were revisited on a regular basis with yearly clinical and MRI investigations.

**Results:**

Pat mean age was 61.5 years (mean deviation 8.2), mean tumor diameter was 13.6 mm (3.4) largest area was 150 mm2 (79.3), mean operation time was 255.2 minutes (48.7). In all but one case the facial nerve could be preserved with normal function, in one pat the postop result was HB 3 with improvement to 2. The acoustic nerve was anatomically preserved in 8/10 cases. Hearing was preserved at the same level as preoperatively in 7/10 cases. Tumor resection was complete in 9/10 patients. In one pat a small contrast enhancing area is under control and stable. There were no surgical complications as CSF fistula or infections.

**Conclusion:**

Exclusively endoscopic resection of CPA tumors is feasible. Results are comparable to microscopic resections. Visualisation in the posterior fossa is excellent, especially in the rostral area of the lesion and in the IAC. Retractors are not always necessary. The size of the approch may be reduced and tailored to the size of the lesion. It remains open if in larger tumors this technique is also beneficial. Problems may occur with severe bleedings in the operating field.

